# The force-generation capacity of the tibialis anterior muscle at different muscle–tendon lengths depends on its motor unit contractile properties

**DOI:** 10.1007/s00421-021-04829-8

**Published:** 2021-10-22

**Authors:** Alessandro Cudicio, Eduardo Martinez-Valdes, Marta Cogliati, Claudio Orizio, Francesco Negro

**Affiliations:** 1 Department of Clinical and Experimental Sciences, Università degli Studi di Brescia, Brescia, Italy; 2 Centre of Precision Rehabilitation for Spinal Pain (CPR Spine), School of Sport, Exercise and Rehabilitation Sciences, University of Birmingham, Birmingham, UK

**Keywords:** Muscle–tendon length, Joint position, Motor unit, Discharge rate, Recruitment, HDEMG

## Abstract

**Purpose:**

Muscle–tendon length can influence central and peripheral motor unit (MU) characteristics, but their interplay is unknown. This study aims to explain the effect of muscle length on MU firing and contractile properties by applying deconvolution of high-density surface EMG (HDEMG), and torque signals on the same MUs followed at different lengths during voluntary contractions.

**Methods:**

Fourteen participants performed isometric ankle dorsiflexion at 10% and 20% of the maximal voluntary torque (MVC) at short, optimal, and long muscle lengths (90°, 110°, and 130° ankle angles, respectively). HDEMG signals were recorded from the tibialis anterior, and MUs were tracked by cross-correlation of MU action potentials across ankle angles and torques. Torque twitch profiles were estimated using model-based deconvolution of the torque signal based on composite MU spike trains.

**Results:**

Mean discharge rate of matched motor units was similar across all muscle lengths (*P* = 0.975). Interestingly, the increase in mean discharge rate of MUs matched from 10 to 20% MVC force levels at the same ankle angle was smaller at 110° compared with the other two ankle positions (*P* = 0.003), and the phenomenon was explained by a greater increase in twitch torque at 110° compared to the shortened and lengthened positions (*P* = 0.002). This result was confirmed by the deconvolution of electrically evoked contractions at different stimulation frequencies and muscle–tendon lengths.

**Conclusion:**

Higher variations in MU twitch torque at optimal muscle lengths likely explain the greater force-generation capacity of muscles in this position.

## Introduction

The amount of force that is generated by a muscle and its rate of change in force depend on both its length and shortening velocity (Askew and Marsh 1998; Hager et al. 2020). When considering isometric voluntary contractions, a muscle can generate its highest force at its optimal length. Thus, a muscle’s maximal force output can be significantly reduced when shortened or stretched in relation to its optimal length (Haffajee et al. 1972; Christova et al. 1998; Del Valle and Thomas 2004). Although previous literature suggested that this phenomenon is mainly related to changes in peripheral properties such as the amount of actin–myosin overlap and Ca^2+^ sensitivity (see Rassier et al. 1999 for a review), other studies have shown that both neural (central) and peripheral factors can be responsible for the differences in force generation across varying muscle lengths (Vander Linden et al. 1991; Bigland-Ritchie et al. 1992; Kennedy and Cresswell 2001; Pasquet et al. 2005). For instance, Marsh et al. (1981) and Sale et al. (1982) previously demonstrated that the optimal muscle–tendon length depends on the activation level (Holt and Azizi 2014). These authors showed that increasing rates of repeated electrical stimulations delivered to the motor point could shift the muscle length at which maximal force can be produced. For these reasons, an increase in motor unit discharge rate (DR)/recruitment is necessary to exert the same absolute forces at lengths different than the optimal. Nevertheless, studies that have analyzed motor unit activity at different muscle lengths have reported conflicting results, with some studies showing different (Tax et al. 1990; Ballantyne et al. 1993; Christova et al. 1998; Kennedy and Cresswell 2001; Altenburg et al. 2008; Lauber et al. 2014; Kirk and Rice 2017) or similar (Bigland-Ritchie et al. 1992; Del Valle and Thomas 2004; Altenburg et al. 2009; Hali et al. 2020, 2021) changes in motor unit firing properties (i.e., DR and recruitment threshold) at multiple joint angles. Although some of these results can be explained by differences between bi-articular and monoarticular muscle’s mechanical properties (Kennedy and Cresswell 2001), many studies have even reported contrasting results on the same monoarticular muscle [i.e., tibialis anterior (TA) (Vander Linden et al. 1991; Bigland-Ritchie et al. 1992; Pasquet et al. 2005)]*.* Two potential issues can explain these diverse findings: first, the identification of the same motor units in contractions at different lengths can be considerably challenging due to the variation of motor unit action potential (MUAP) shapes of intramuscular recordings (Dumitru et al. 1999); second, the relation between motor unit DR and twitch temporal profiles can vary across different muscle lengths and activation levels (Bigland-Ritchie et al. 1992; Christova et al. 1998). On the one hand, long muscle lengths show evoked single-twitch profiles of longer duration and higher amplitude compared to shorter muscle lengths (Marsh et al. 1981; Bigland-Ritchie et al. 1992; Christova et al. 1998), and for these reasons, they generate the highest force at low stimulation rates (Marsh et al. 1981; Holt and Azizi 2014). On the other hand, at higher stimulation rates, the optimal length shifts towards shorter lengths. Consequently, it is reasonable to assume that, during voluntary contractions at specific submaximal force levels, the average DR of the active motor units will adapt in relation to the variations in twitch forces at different muscle–tendon lengths (Inglis et al. 2011). Nevertheless, the effect of changes in contractile properties has only been associated with changes in the overall muscle twitch force during electrically evoked contractions at moderate to high rates. The impact of compound and average motor unit twitch force during voluntary and electrically stimulated contractions at different muscle lengths (fused twitches) is yet to be determined.

To improve our understanding of the effects of different ankle angles on motor unit properties, techniques enabling accurate tracking of the same motor units across a wide range of ankle angles and forces are necessary. In addition, both central (i.e., DR, recruitment threshold) and peripheral (i.e., motor unit twitch force) motor unit properties need to be assessed together to understand better how changes in muscle properties influence neural activity and vice-versa. Recently, high-density surface electromyography (HDEMG) decomposition techniques have shown the ability to compensate for MUAP non-stationarities and extract individual motor unit activity during contractions at varying muscle lengths (Glaser and Holobar 2019). Moreover, due to their larger recording area, tens of motor units can be identified during the contractions, allowing a more robust estimation of the average motor unit twitch contraction force (Negro and Orizio 2017). The knowledge obtained with these methods can provide new information about the strategies employed by the nervous system to control force at different muscle lengths. Therefore, with the combination of HDEMG tracking (Martinez-Valdes et al. 2017) and advanced twitch estimation techniques (Negro and Orizio 2017), this study aimed to understand the effect of TA muscle length and torque on motor unit firing, recruitment, and contractile properties at different ankle angles. First, we verified previous results on the relation between mean discharge rate and recruitment parameters of the same motor units at different ankle angles. Second, we hypothesized that contractile motor unit properties would determine the higher force-generation capacity at the optimal ankle angle. We confirmed this hypothesis using both voluntary and electrically evoked contractions at moderate force levels.

## Materials and methods

### Participants and ethical approval

Fourteen young, healthy subjects (1 female and 13 males, age 26 ± 3 years, weight 76 ± 10 kg, height 178 ± 9 cm) without neurological or orthopedic disorders gave their informed consent to participate in this study. The local Ethical Research Committee approved the proposed experimental design (CEIOC authorization: NP2490) in accordance with the Declaration of Helsinki (2004). Participants were asked to refrain from strenuous exercise 48 h before testing and avoid caffeine consumption 24 h prior to testing.

### Task

**Fig. 1:**
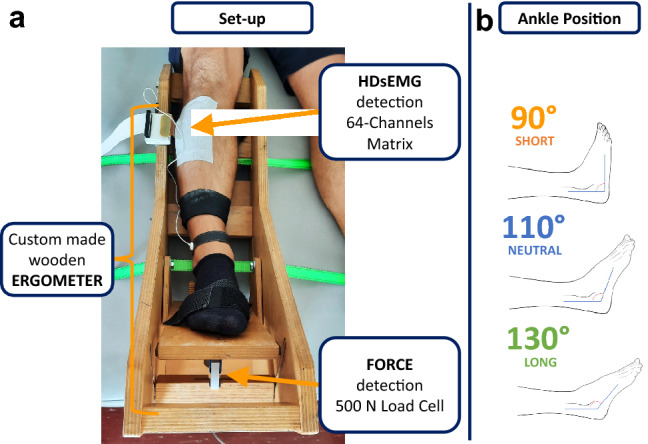
Schematic representation of the experimental setup (**a**). The right leg of the subject was fastened to the custom-made wooden ergometer. The foot was ensured to the support with Velcro straps. The HDsEMG was recorded with one matrix applied to the proximal part of the TA muscle belly. The torque was detected with a 500 N load cell. This particular ergometer allowed to set the ankle angle in different positions to achieve three muscle–tendon lengths: short, optimal, and long, as reported on the right (**b**)

The participants laid back in a semi-seated position on a plinth with the right leg placed and fixed in a custom-made wooden ergometer. The sole of the right foot was fixed to an adjustable support, and the ankle was tied with a strap (Fig. 1a). The knee was completely extended (knee angle 180°), and the back of the subjects rested against the seatback with a hip flexion angle of 30°. The subjects were instructed to keep their left leg straight and relaxed on the plinth, on the side of the ergometer. The foot support of the ergometer could be set at any dorsiflexion angle in the full range of ankle dorsi-plantar flexion. The foot support was connected to a 500 N load cell (SM-500 N, Interface, Arizona, USA) to collect ankle dorsiflexion torque. All participants were asked to exert isometric ankle dorsiflexion torque at three different ankle angles (90°, 110°, and 130°, which correspond to 0°, 20°, and 40° of plantar flexion, respectively) (Fig. 1b). These angles were selected as they represent lengths where the TA muscle is shortened (90°), placed at optimal resting ankle angle (110°), and stretched (130°). A screen in front of the participant placed at a distance of approximately 1 m showed the torque target that had to be reached (red line) and the dorsiflexion torque feedback (black line). All participants underwent a brief warm-up and training session before the beginning of the experiment. At each dorsiflexion angle, the participants were asked to perform three maximal voluntary contractions (MVC) lasting 3 s. Each of these MVCs was separated by 2 min of rest. If the difference between the MVCs was > 10%, additional trials were performed until this criterion was met. The highest of the three MVC trials was used as a reference for the submaximal torque targets. At each angle, it was required to perform two isometric contractions: one at 10%MVC and another at 20%MVC. The contractions were separated by 2 min of rest and were performed randomly. The trapezoidal-shaped target displayed on the monitor was reached at a rate of 5%MVC/s and maintained for 40 s; then, the torque decreasing rate was set at 5%MVC/s. The relatively long steady part of the target was selected to provide reliable motor unit decompositions and twitch estimations.

In addition, in a subgroup of five participants, electrically evoked contractions at frequencies of 5, 10, 15, and 20 Hz at the same three ankle positions (short, optimal, and long) performed on experiment 1 were recorded. In this experiment, the common peroneal nerve of the participants was electrically stimulated to evoke an involuntary contraction of the TA at the three muscle–tendon lengths. The cathode (cup electrode with a radius of 1 cm, Spes Medica s.r.l., Genova (GE), Italy) was filled with conductive paste (AC cream, Spes Medica s.r.l., Genova (GE), Italy), and it was applied posterior inferior to the head of the fibula. The anode (rectangular electrode 5 × 10 cm, UltraStim, Axelgaard Manufacturing CO., LTD, CA, USA) was applied on the opposite side of the knee. First, a train at 1 Hz frequency and increasing amplitudes was delivered for 70 s. This procedure allowed to identify the stimulus amplitude eliciting the largest stable torque response defined as the amplitude threshold. Then, the stimulation protocol was designed as follows: for each muscle–tendon length, four 15-s trains with frequencies of 5–10–15–20 Hz were delivered. Each train was separated from the subsequent by at least 90 s of resting period. The amplitude of the stimulus was set at 120% of the amplitude threshold, and its duration was 0.2 ms. The stimulation trains were not periodic, but included a significant amount of variability in order to generate a more physiological response and obtain a unique solution for the inverse deconvolution problem (Negro et al. 2014). The imposed variability generated a coefficient of variation for the inter-stimulus intervals of approximately 90%.

### Data acquisition

After the participant’s skin was shaved and cleaned with abrasive paste (EVERI, Spes Medica s.r.l., Genova (GE), Italy) and water, one 64-channel matrix (8 mm inter-electrode distance, OT Bioelettronica s.r.l., Torino (TO), Italy) was placed longitudinally on the TA muscle belly. For a better fitting on the skin, an adhesive foam filled with conductive paste (AC cream, Spes Medica s.r.l., Genova (GE), Italy) was applied to the matrix. Reference electrodes were positioned on the right wrist and right ankle. An EMG-USB2+ amplifier (12-bit analog to digital converter, 3 dB, bandwidth 10–500 Hz; OT Bioelettronica s.r.l., Torino (TO), Italy) was used to record HDEMG and torque signals with a sampling frequency of 2048 Hz. HDsEMG signals were recorded in monopolar mode and the EMG signal gain was verified before each task, to maximize signal resolution and avoid saturation. OT-BioLab software (OT Bioelettronica s.r.l., Torino, Italy) was used to record both HDEMG and torque signals.

### Data analysis

**Fig. 2:**
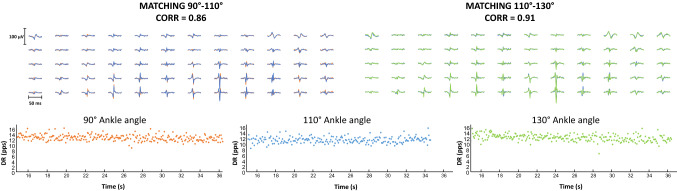
Example of motor unit tracking across the three ankle angles. On the top part, the matched motor unit action potentials across ankle angles are shown between 90° (orange line) and 110° (blue line) with cross-correlation of 0.86 and between 110° and 130° (green line) with cross-correlation of 0.91. On the bottom part, the similar instantaneous discharge rate profiles of the same motor unit for the three ankle angles are shown

After exclusion of EMG channels with poor signal quality, the convolutive blind-source separation algorithm was used to identify single motor units (MU) from the HDsEMG signal (Negro et al. 2016). Motor unit discharge times of single motor units were checked, and any missing or non-physiological inter-spike intervals (i.e., < 33.3 ms) were edited manually. Following the addition of missing firings and/or the removal of non-physiological discharges, motor unit spike trains were re-estimated as described previously (Hassan et al. 2020; Martinez‐Valdes et al. 2020a, b; Afsharipour et al. 2020) and as proposed in other studies (Del Vecchio et al. 2020; Hug et al. 2021). Following the editing and correction of motor unit spike trains, each motor unit was tracked and matched across the different ankle angles and different torque levels by cross-correlation of the 2D representation of the MUAP waveforms (Martinez-Valdes et al. 2017). To our knowledge, this is the first study tracking relatively large samples of motor units at different muscular lengths. Since large variations in muscle length can potentially influence MUAP shapes, we first assessed whether motor units could be tracked across closer angles. For this purpose, we assessed the tracking accuracy of motor units at an ankle angle of 90°, 100°, 110°, 120° and 130° in seven individuals prior to the main study. From this pilot data, we could confirm that motor units could be tracked across the full range of motion (average cross-correlation across angles: 0.92 ± 0.02); however, as the cross-correlation of these motor units was slightly reduced between 90°, 110° and 130°, we decreased the matching threshold to 0.70 to assume that the MUAPs belonged to the same motor unit across the three plantar-flexion angles. A representative example of the tracking procedure can be seen in Fig. 2. The upper part of Fig. 2 shows the MUAPs belonging to the same motor units at different ankle angles. The instantaneous DR is shown in the lower part of Fig. 2. Mean DR and the coefficient of variation of the inter-spike interval were calculated for each tracked motor unit in the steady torque part of the contraction. The DR at recruitment and de-recruitment, and recruitment and de-recruitment thresholds (normalized to %MVC and in absolute Nm values) were also calculated. The recruitment and de-recruitment threshold were defined as the dorsiflexion torque at the time when the motor unit began and stopped firing action potentials. DR at recruitment and de-recruitment was calculated with the first six and last six motor unit firings, respectively (Martinez‐Valdes et al. 2020a; b). Each of these variables was calculated for each torque level and angle independently. The variation in discharge rate per %MVC (∆ discharge rate) for motor units tracked at different torques (10% and 20% MVC) was quantified at each ankle angle and averaged within subjects.

Motor unit twitch contraction torque at each submaximal torque level and angle was determined with a recent technique based on the deconvolution of the torque signal using the discharge times of the identified motor units (Negro and Orizio 2017). In the electrically evoked contractions, we used a similar approach but with a more comprehensive twitch model (Raikova et al. 2007). Briefly, we performed an exhaustive search of a relatively large range of twitch parameters to find the best reconstruction of the force profile (minimum squared Euclidean distance between the detrended original and reconstructed force traces). After performing the estimation, we calculated the variation in twitch area across force levels for the voluntary experiment. To quantify the variation of the twitch area of the matched motor unit across the torque level, the ratio was calculated using the following formula: (twitch_area_20%MVC − twitch_area_10%MVC)/twitch_area_10%MVC * 100. In the experiment with electrical stimulation, the total variation was calculated as the slope coefficient of the linear regression across frequencies of stimulation at each ankle position.

More details about these twitch estimation techniques are reported in Appendix.

### Statistical analysis

All results are expressed as mean and standard deviation unless otherwise stated. All variables were tested for normality using the Shapiro–Wilk test. The assumption of sphericity was checked by the Mauchly test, and if violated, the Greenhouse–Geisser correction was made to the degrees of freedom. Statistical significance was set at *P* < 0.05. Differences in MVC across the three angles were made with a one-way repeated-measures ANOVA. Variations in ∆ discharge rate, % variation in twitch area between 10 and 20% MVC, and area slope of stimulated twitches across the three angles were also compared with one-way repeated-measures ANOVA. All other motor unit parameters were compared with two-way repeated-measures ANOVA with factors of angle (90°, 110°, and 130°) and torque level (10% and 20% MVC). Pairwise comparisons were made with Bonferroni post hoc tests when ANOVA was significant.

## Results

### Motor unit decomposition and tracking

A total of 1848 motor units were identified across the different torque levels and ankle angles in the TA, with an average of 66 ± 27 motor units per subject (across all angles and torques combined). When considering each contraction independently, 22 ± 10, 23 ± 8 and 21 ± 10 motor units per subject were identified per subject at 90°, 110°, and 130°, respectively, at both 10% and 20% MVC. Among these identified motor units, a total of 152 and 186 could be reliably tracked by two-dimensional cross-correlation of the MUAPs across the three ankle angles at 10% and 20% of MVC, respectively. The average number of tracked motor units per subject was 11 ± 7 and 13 ± 8 at 10% and 20% of MVC, respectively. An example of motor unit tracking is reported in Fig. 2. The average cross-correlation coefficient of the tracked motor units at 10% MVC ranged between 0.70 and 0.93 (mean 0.85 ± 0.06), and at 20% MVC ranged between 0.78 and 0.93 (mean 0.87 ± 0.04).

### Torque

**Fig. 3:**
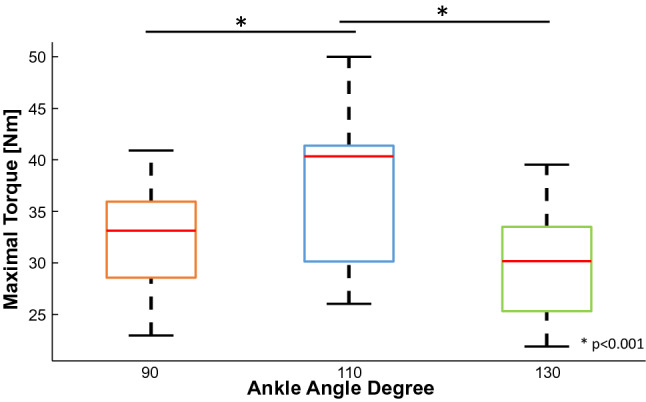
Maximal voluntary contraction. The MVC estimated at 110° ankle angle, in blue, (mean = 37.5 ± 7.1 Nm) was statistically higher in respect to the values at 90°, in orange, (mean = 32.3 ± 5.5 Nm) and 130°, in green, (mean = 30.1 ± 5.3 Nm) (*P* ≤ 0.001). The three boxplots represent the maximum, minimum, and median values at each ankle angle

The MVC at the optimal length was 13.7% and 19.7% higher compared to the one achieved at the short (*P* = 0.001) and long length (*P* < 0.001) of the TA, respectively (Fig. 3). No statistical difference was found between the MVC values at 90° and 130°. The mean coefficient of variation of torque at 10% MVC was 2.8 ± 1.3, 2.1 ± 0.9 and 2.5 ± 0.6%, and at 20% MVC was 2.2 ± 1.3, 2.1 ± 0.7 and 2.0 ± 0.6 for the 90°, 110°, and 130° ankle angles, respectively (*P* = 0.040, $\eta_{{\text{p}}}^{2}$ = 0.286).

### Motor unit firing properties

Mean motor unit DR from the tracked motor units increased with torque level (torque effect: *P* < 0.001, $\eta_{{\text{p}}}^{2}$ = 0.847), and this increase was similar across all joint angles (angle effect: *P* = 0.975, $\eta_{{\text{p}}}^{2}$ = 0.002). DR at recruitment also showed similar changes across torques (torque effect: *P* = 0.006, $\eta_{{\text{p}}}^{2}$ = 0.456) but no differences across angles (*P* = 0.859, $\eta_{{\text{p}}}^{2}$ = 0.012). Normalized recruitment threshold increased both with torque (torque effect: *P* < 0.001, $\eta_{{\text{p}}}^{2}$ = 0.851) and angle (angle effect: *P* = 0.041, $\eta_{{\text{p}}}^{2}$ = 0.218). The coefficient of variation for the inter-spike intervals slightly decreased with the increasing of torque (torque effect: p = 0.047, $\eta_{{\text{p}}}^{2}$ = 0.271) and did not change significantly across the different joint angles (angle effect: *P* = 0.648, $\eta_{{\text{p}}}^{2}$ = 0.033). Mean values for all these parameters are reported in Table 1.

**Table 1:** Summary of mean discharge rate, recruitment discharge rate, recruitment torque, and inter-spike interval for each ankle angle and torque level

Ankle angle (°)	Torque level (%MVC)	Mean DR (pps)**	Recruitment DR (pps)*	Recruitment torque (%mvc)**^†^	Inter spike interval CoV (pps)*
90	10	10.8 ± 1.7	9.0 ± 1.9	4.0 ± 1.7	13.8 ± 3.5
	20	12.4 ± 1.7	10.1 ± 1.7	10.1 ± 4.0	13.9 ± 3.7
110	10	10.9 ± 1.5	9.4 ± 2.3	4.0 ± 2.5	13.1 ± 2.8
	20	12.2 ± 1.7	10.0 ± 2.2	10.8 ± 4.3	15.8 ± 3.5
130	10	10.8 ± 1.5	9.2 ± 2.1	5.8 ± 2.6	14.0 ± 3.1
	20	12.2 ± 1.8	10.3 ± 2.1	11.9 ± 3.9	15.1 ± 4.3

Mean ± SD; the asterisk indicates statistical difference across torque level (*P < 0.05, **P < 0.001) and the cross across angle (^†^*P* < 0.05)

**Fig. 4:**
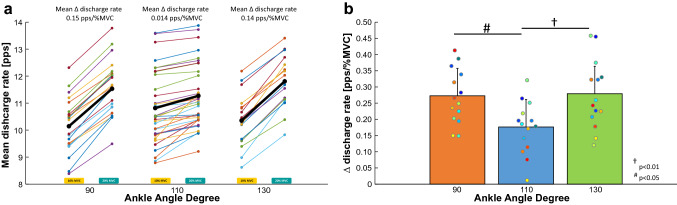
**a** The discharge rates of the matched motor units (across the two force levels) of a representative subject are reported with dots of the same color and connected with a solid line (left 10%MVC and right 20%MVC). A black line shows the mean variation (∆ discharge rate) of the matched motor units for the three ankle angles. **b** The bar plot shows the mean and standard deviation of the ∆ discharge rate across the three positions, 90° (orange), 110° (blue), and 130° (green). Statistical differences between 110° and 90° and between 110° and 130° ankle angle were reported (*P* < 0.05). Individual subject values are reported with dots of the same color

Figure 4a shows the change in mean DR for all motor units tracked across the two force levels at each ankle angle for a representative subject. Across all subjects, we found a significant difference of ∆ DR within angles (*P* = 0.003, $\eta_{{\text{p}}}^{2}$ = 0.355, Fig. 4b) with the joint angle at 110° showing a significantly smaller variation compared to joint angles at 90° and 130° (Fig. 4b).

### Motor unit contractile properties

**Fig. 5:**
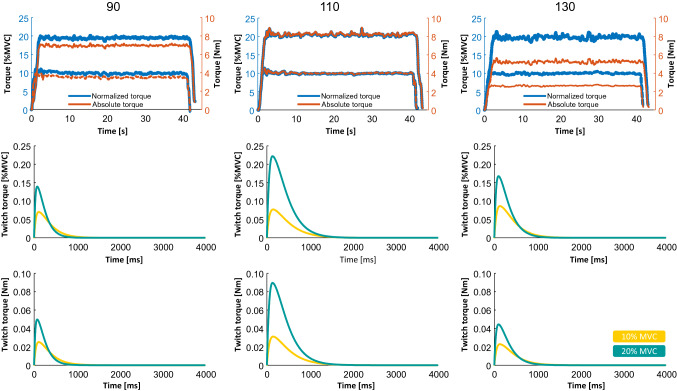
Representative example of motor unit twitch estimation across ankle angles. Top: the torque profiles (absolute values in orange, relative values in blue) are shown. Twitch estimations are shown in relative (middle plots) and absolute (bottom plots) units. Estimations performed at 10% MVC are shown as yellow and cyan lines at 20% MVC

**Fig. 6:**
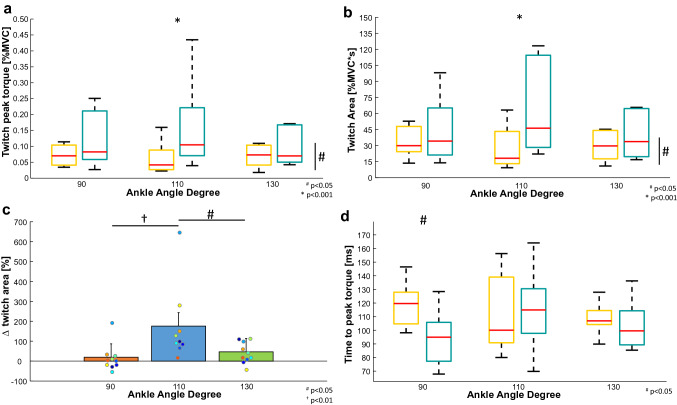
Twitch peak (**a**), twitch area (**b**), twitch area variation (**c**), and time to peak (**d**) mean values and standard deviations of the identified motor units are reported. The redline of the boxplots represents the median value at 10% MVC (yellow box) and 20% (cyan box) in the three conditions: 90° short muscle on the left, 110° neutral position in the middle, and 130° stretched muscle on the right. The bottom and the top of the box are respectively the 25th and the 75th percentiles. The dashed lines indicate the minimum and the maximum value. In **a** and **b**, the mean values were statistically different at 110° (*P* < 0.001). In (**c)**, statistical differences between 110° (blue bar) and 90° (orange bar, *P* < 0.01) and between 110° and 130° (green bar, *P* < 0.05) ankle angle are shown. In **d**, there was a statistical difference between values at 90° and the other two ankle angles (*P* < 0.05)

**Fig. 7:**
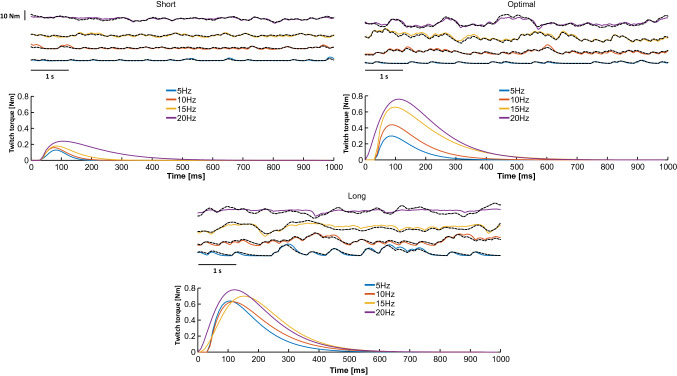
Representative example of twitch estimation across muscle lengths at different frequencies of stimulation. Torque profiles on the top and twitch torque estimations on the bottom are shown. Blue, red, yellow, and purple lines represent 5 Hz, 10 Hz, 15 Hz, and 20 Hz frequencies of electrical stimulation, respectively

**Fig. 8:**
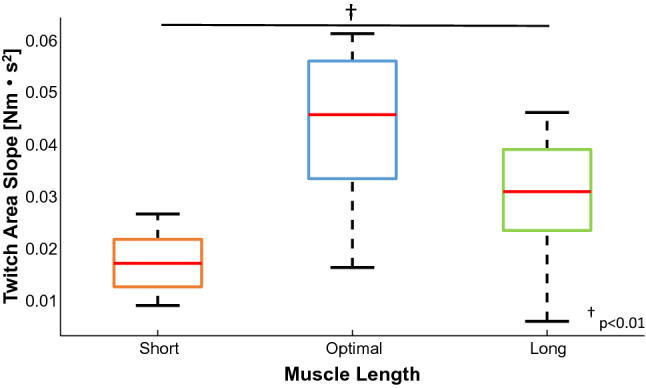
The bar plot shows the mean and standard deviation of the twitch area slope across the three positions for the stimulated contractions, short (orange), optimal (blue), and long (green) length. The statistical difference across muscle length is reported (*P* < 0.01)

Changes in motor unit twitch profile are shown for a representative subject in Fig. 5. In this participant, twitch profiles remained similar across all ankle angles at 10% MVC (yellow profiles) but differed at 20% MVC (light-blue profiles), as both motor unit twitch peaks and areas were larger at the optimal muscle length (110°) compared to shorter (90°) and longer (130°) muscle lengths. These results were confirmed at a group level as we observed that twitch peak and area showed a significantly higher increase at 20% MVC when the joint was positioned at 110° compared to 90° and 130° (angle*torque interaction: *P* = 0.03, $\eta_{{\text{p}}}^{2}$ = 0.420 and *P* = 0.008, $\eta_{{\text{p}}}^{2}$ = 0.413, for twitch peak and twitch area, respectively, Fig. 6a and b), with greater variation in twitch area values between 10 and 20% MVC at 110° (Fig. 6c) (angle effect: *P* = 0.002, $\eta_{{\text{p}}}^{2}$ = 0.479). There was a significant decrease in the time to peak at 20% MVC at shorter lengths only (90°, Fig. 6d) (angle*torque interaction: *P* = 0.038, $\eta_{{\text{p}}}^{2}$ = 0.30). Finally, changes in the twitch profile in the electrical stimulation protocol can be seen for a representative subject in Fig. 7. In this participant, twitch profiles remained similar across all stimulation frequencies in both short and long positions, but they show larger variations at the optimal position from 5 to 20 Hz, as only in this position, twitch force increased significantly with stimulation frequency. These results confirm that changes in twitch area across frequencies of activation depend on muscle length (length effect: *P* = 0.006, $\eta_{{\text{p}}}^{2}$ = 0.722) as reported in Fig. 8 for the whole group of participants.

### Interference electromyography

Root mean square EMG amplitude in absolute units was significantly higher at 20% compared to 10% MVC, but was similar across all angles (torque effect: *P* < 0.001, $\eta_{{\text{p}}}^{2}$ = 0.855, angle effect: *P* = 0.844, $\eta_{{\text{p}}}^{2}$ = 0.013) showing values at 10% MVC of 107 ± 5 1, 108 ± 38 and 101 ± 3 0 μV at 90°, 110° and 130°, respectively, and values of 170 ± 68, 171 ± 54 and 166 ± 51 μV at 90°, 110°, and 130°, at 20% MVC, respectively.

## Discussion

The study aimed to assess TA motor unit firing and contractile properties over a wide range of TA lengths at two torque levels (10 and 20% MVC) by tracking the same motor units across multiple ankle angles during dorsiflexion. The results showed that muscle–tendon length changes did not influence mean DR, but affected the variation in DR with increasing torque across the different joint angles. Thus, when the muscle is positioned at its optimal length, it requires a lower increase in firing frequency to attain a higher torque level compared to a more shortened or lengthened position. Moreover, we could demonstrate that this was related to differences in the average motor unit twitch torque area since the variation of this parameter was larger between 10 and 20% MVC at the optimal length compared to 90° and 130°. In addition, we were able to confirm this phenomenon using electrically stimulated twitches. Taken together, our results suggest that modulations in motor unit firing across different muscle lengths are regulated by motor unit contractile properties depending on the mean activation level of the muscle.

### The effect of muscle length on motor unit firing properties

Previous studies assessing changes in motor unit firing properties have reported mixed results. The wide variety of joint positions examined and the assessment of absolute versus normalized to the MVC forces (according to the MVC attained at each joint angle) make the comparison of results across studies challenging. For example, some authors considered the “neutral position” (i.e., anatomical position of 90°) of the investigated joint as the reference position (Vander Linden et al. 1991; Bigland-Ritchie et al. 1992; Pasquet et al. 2005) while others considered the joint’s optimal length (i.e., position where maximal force can be exerted) as the reference position (Marsh et al. 1981; Altenburg et al. 2009; Soucie et al. 2011). Moreover, some investigators have extracted motor unit properties during isometric contractions at different muscle lengths using the same absolute torque (Vander Linden et al. 1991; Kennedy and Cresswell 2001), while the other authors have normalized the torque according to the MVC force achieved at each investigated muscle length (Marsh et al. 1981; Bigland-Ritchie et al. 1992; Pasquet et al. 2005). Moreover, previous intramuscular EMG methods used in these investigations also showed many difficulties in tracking the same motor units across different joint angles, and this could have induced the assessment of different populations of motor units across different muscle lengths. Therefore, it is not surprising that previous studies have reported differences or no differences in mean DR at different lengths (Vander Linden et al. 1991; Bigland-Ritchie et al. 1992; Christova et al. 1998; Pasquet et al. 2005; Altenburg et al. 2008, 2009). To overcome these issues, first, we normalized the target torque exerted by the participants according to each muscle–tendon length MVC, as an attempt to standardize the target torque levels according to each muscle’s length maximal torque output. Second, we attempted to overcome the difficulties of intramuscular EMG recordings in tracking the same motor units across joint angles by employing motor unit matching using HDEMG recordings.

As a methodological consideration, when motor unit properties are assessed across different joint angles, the relative position of the detection points in respect to the muscle fibers changes, and due to the high selectivity of the intramuscular EMG recordings, the shape of the action potentials can vary dramatically with variations in muscle–tendon length. This problem may be mitigated using HDEMG recordings thanks to the low selectivity and the high spatial resolution of these systems. With this methodology, we managed to track a relatively large number of motor units in the TA muscle across a wide range of motion (40°). To achieve such high tracking performance, we estimated the average variation in MUAP shapes across ankle angles, and subsequently, compensated for this effect. Specifically, in a subset of seven subjects, we first divided the range of ankle positions in small steps of 10°, and then, we tracked the same motor units across wider ankle angles (20°). In this way, we could confirm that the tracking across intervals of 20° was still applicable by decreasing the threshold of the two-dimensional correlation of the MUAPs at different muscle–tendon lengths. Using this methodology, we were able to track an average of 12 ± 7 motor units per subject across the three ankle angles (90, 110, and 130). However, it is important to underline that we placed the matrix of electrodes in the proximal portion of the tibialis anterior muscle, and our results may not be easily generalized to more distal parts where regional shifts and variations in propagation characteristics of the action potentials may become more evident at different muscle lengths.

With this accurate tracking criteria, we found that the mean DR and DR at recruitment were similar across different ankle angles for both 10 and 20% MVC contractions. Recruitment thresholds were found lower at the shortest ankle angles compared to the longest, but only when using normalized torque values. No change in any motor unit parameter at de-recruitment was found. These findings align with studies that have used intramuscular recordings in TA muscle and have employed normalized forces across different lengths. For instance, Pasquet et al. (2005) tracked a small sample of motor units at short and long muscle lengths in a narrower range of motion (20°) and reported similar DRs across angles and increased normalized recruitment threshold force (%MVC) at long muscle lengths. Moreover, Bigland-Ritchie et al. (1992) also found similar DRs between 0° and 15° of dorsiflexion in untracked motor units. Similar results have also been reported for other monoarticular muscles such as the Vastus Lateralis (Altenburg et al. 2009). Nevertheless, results can vary in other bi-articular muscles such as the medial gastrocnemius, where changes in the position of proximal (knee) and distal (ankle) joints can induce variations in DR and recruitment threshold (Kennedy and Cresswell 2001; Lauber et al. 2014). Therefore, it is possible that the findings presented in the current study are only applicable to monoarticular muscles.

The maintained DR across ankle plantar-flexion angles can be explained by differences in twitch force and motor unit recruitment at different joint angles. Recent research has shown that changes in twitch force can affect motor unit firing frequency; therefore, low twitch forces can be compensated by increasing DR and high twitch forces, require less DR to maintain the required force level (Inglis et al. 2011; Martinez‐Valdes et al. 2020a, b; Cogliati et al. 2020a). Considering this observation, previous studies have reported that whole-muscle stimulation at low rates generates the maximal force at longer muscle lengths. On the other hand, peak twitch torque is maximal during high rate stimulation at the optimal muscle length (Marsh et al. 1981). Therefore, the fact that we observed similar DRs across all the assessed joint angles despite the lower peak torque values exerted at both shortened and lengthened positions shows that the force-generation capacity of the muscle is higher at these average activation rates at the optimal length.

### Variations in mean discharge rate and twitch area across torque levels and muscle lengths

To understand the neural factors allowing the generation of the highest torque at the optimal length, we estimated the discharge rate properties of matched motor units across the two target torque levels (10% vs 20% MVC) at each ankle angle. Using this approach, we could observe a significant increase in DR when the target torque changed from 10 to 20% MVC (Fig. 4a). In particular, when we considered the variation ∆ discharge rate across the two torque levels, we could observe that DR increased less at optimal length compared to the lengthened and shortened positions (Fig. 4b). The reason for requiring less increasing of discharge rate from lower to higher torque levels in the optimal condition could be related to the muscle’s contractile properties, which can influence the discharge rate required to reach a specific torque level. Previous studies have shown that the relation between generated torque and muscle–tendon length during electrically stimulated contractions is approximately linear for low stimulation intensities but becomes nonlinear at higher stimulation rates (Marsh et al. 1981). In particular, the maximal torque generation can be reached at a muscle–tendon length similar to the length that provides the maximal voluntary force using stimulation rates that produce a significant fusion of individual twitches. This phenomenon demonstrates that active contractile properties of muscles may play an important role in the generation of force at different muscle–tendon lengths.

In our study, we estimated the contractile properties of the ensemble of the decomposed motor units using a recently proposed technique for the deconvolution of the force signal based on the estimated neural drive information (Negro and Orizio 2017). Our results showed that the estimated average twitch profile area and twitch peak increased significantly more between 10 and 20% MVC at the optimal length compared to the shortened and lengthened positions. The variation in contractile motor unit properties agreed with the smaller change in DR of the matched units identified across force levels at the optimal length. Furthermore, to confirm these latter results, we evaluated, in a subgroup of five participants, the twitch profiles evoked by repeated electrical stimulation. Several studies tried to explain the role of peripheral motor unit properties in several muscles, evoking a single twitch across different muscle–tendon lengths (Winegard et al. 1997; Mela et al. 2001; Kluka et al. 2015; Behrens et al. 2019; Hali et al. 2021). However, the characteristics of individual twitches do not reflect the contractile properties of a muscle during voluntary contractions. For these reasons, previous studies have shown significant differences in the force–stimulation frequency relations across different muscle–tendon lengths (Marsh et al. 1981; Mela et al. 2001). Consequently, the evaluation of the characteristics of compound twitches extracted from evoked tetanus could be useful to understand the relation between muscle–tendon length and motor unit contractile properties. In this study, we were able to deconvolve individual force twitches during repeated stimulation (Raikova et al. 2007) of the tibialis anterior at short, optimal and long muscle–tendon lengths. Interestingly, we found that the areas of the decomposed twitches only varied at the optimal length (twitches increased with stimulation frequency) and remained constant at short and long muscle–tendon lengths. In particular, the slope of the regression of the twitch torque area across stimulation rates was dependent on muscle–tendon length (*P* < 0.01) (Fig. 8).

According to the results of Marsh et al. (1981), greater increases in toque output are expected at higher stimulation frequencies in the optimal position. This observation supports the idea that motor unit peripheral properties during repeated activation play a key role in the force generation at different muscle–tendon lengths. These results are in line with what was previously demonstrated in animal models. For example, Rakoczy et al. (2020) showed that the decrease in transitory force output was greater in the optimal position compared to the more stretched or shortened muscle. In other words, while passive contractile properties change progressively with muscle–tendon length (Marsh et al. 1981), the active contractile properties of the motor units may be efficiently tuned to generate more force at the optimal length. In this view, the optimal length depends on the overall activation level of the muscle because motor unit twitch fusion will be higher and will require less variation in average DRs across increasing force levels. Considering a maximal rate of change in discharge rate of the alpha motor neurons in voluntary contractions, the nonlinear increase in force capacity will provide the maximal rate of force development at the optimal length (Hager et al. 2020; Cogliati et al. 2020b). Therefore, the phenomenon provides a simple mechanism to generate maximal absolute force and maximal rate of force development at these lengths. This observation, nevertheless, needs to be confirmed in future studies assessing motor unit discharge behavior and motor unit twitch force in the whole range of force generation at different speeds of contractions. Nevertheless, it is apparent that variations in active motor unit contractile properties are the main determinants for a greater and faster force generation at optimal muscle lengths.

### Potential sensorimotor interactions at different muscle–tendon lengths

The neural control mechanisms responsible for the modulation of motor unit discharge rate at different muscle–tendon lengths is not fully understood. Cortical and subcortical drives (Glover and Baker 2020; Türker 2021) are likely responsible for the adjustments in the neural drive to the tibialis anterior muscle as a function of its length-dependent variations in contractile properties during submaximal isometric contractions. However, peripheral afferent receptors may likely also play an important role in modulating the neural drive to muscle during force variations at different muscle–tendon lengths. In fact, the response of muscle spindle and Golgi tendon organs are well known to be modulated by the characteristics of the motor unit twitch profiles (McKeon and Burke 1983; Proske and Gandevia 2012; Day et al. 2017). Specifically, muscle spindle activity induced by variations in length pauses during contraction tension increase. This results in a decrease of the net excitatory drive to the motor neuron pool of the same muscle. In a similar way, Golgi tendon organs respond to contraction tension increase generating an inhibition on the net excitatory drive to the motor neuron pool of the homonymous muscle. Therefore, changes in contractile properties during sustained contractions at different lengths may indirectly influence the net excitatory drive to the motor neuron pool by modulating the activity of the peripheral afferent receptors. For these reasons, in our study, at the optimal length, the smaller variation in discharge rate across the two force levels may have been induced by a more modest change in net excitatory drive to the motor neuron pool caused by the effect of a larger variation in motor unit twitch profiles on the peripheral afferent activity.

### Limitations

The proposed study has some important limitations that should be taken into account for the correct interpretation of the results. First, we investigated two low-torque levels due to limitations of the technique used to estimate motor unit twitch torque at high force levels. Although we could not investigate the effect of large force tasks on the behavior of motor units at different muscle–tendon lengths, we believe that the selected force levels are relevant and functional for the typical range of forces generated by the TA muscle during everyday tasks (e.g., gait). Second, our technique for the estimation of the average twitch response from a population of motor units is based on a two-parameter model fitting that may converge to several equivalent solutions in terms of least-squared error (local minima). This can limit the interpretation of the results based on the actual peak or time-to-peak values of the fitted twitch profiles. To mitigate this problem, we have included the measure of the overall area of the estimated twitch. Moreover, the results of the torque fitting may also be influenced by the total number of motor units identified across different torques and muscle lengths (Negro and Orizio 2017). To overcome this issue, we employed the same number of motor units (the minimum selected number was five) to estimate twitch torque across torques and angles. With this adjustment, we could obtain reliable estimates of twitch torque from 10 subjects.

## Conclusions

By tracking a relatively large sample of motor units, this study is the first to demonstrate that active motor unit contractile properties influence the rate of change in DR with increasing torque levels across different muscle lengths. The results show that a smaller increase in DR is required at optimal lengths to increase torque due to the potentiation of motor unit twitch force in this position. These findings suggest that active motor unit contractile properties determine the force that can be exerted across different joint angles.

## Data Availability

The data that support the findings of this study are available from the corresponding author upon reasonable request.

## Appendix

The method applied to determine the motor unit contraction torque encompasses a model-based deconvolution of the force signal using the identified discharge times of a population of motor units. Previously, the proposed technique has been validated using simulations and tested on signals recorded during voluntary activation (Negro and Orizio 2017). The results of the simulations showed that the proposed method provides accurate estimates (relative error < 25%) of the main parameters of the average twitch force when the number of identified motor units is between 5 and 15% of the total number of active motor units. Current HDsEMG decomposition methods allow decoding such relative samples from the active motor unit pool (Farina et al. 2016). However, even when this condition is not met, the cited study shows that the estimates provided by the proposed method are anyway always superior to those obtained by the traditional spike-triggered average approach, especially for high motor unit synchronization levels and when a relatively small number of triggers is available. For the voluntary part, in this study, a simplified model of twitch waveform described by only two parameters (peak amplitude and time to peak) was used (Fuglevand et al. 1993). Briefly, a large range of peak amplitude and time-to-peak values were explored for the reconstruction of the torque profile performed by convolution between the sum of the decomposed motor unit spike trains and the modeled twitch waveform instances. Only motor units discharging in the whole segment of 30-s duration were used. For consistency across ankle angles and to limit the bias introduced by the use of a different number of motor units in the calculation, the minimum number of motor units active for each subject was identified and used for the twitch estimation in all force trials of the same subject. The minimum number of motor units that allowed for the calculation was five. For this reason, four subjects were excluded because they did not meet the allowed minimum number of motor units at least in one of the recorded trials. Multiple combinations of composite spike trains (10) with the selected number of motor units were used in each trial. A sliding window of 10 s was used within a segment of 30 s for the calculation. The final estimation was the average twitch calculated across the different combinations and time segments. For each twitch parameter combination, both the re-constructed and the original torque profiles were first detrended to limit the influence of the unidentified motor units. The squared Euclidean distance was used as a measure of similarity between the two signals (the original and the reconstructed torque), and the twitch parameters providing minimum distance were used as the final estimation. In the electrically evoked contractions, we used a similar approach but with a more comprehensive twitch model. In fact, during electrically stimulated contractions, the stimulation train is known, and this helps to find the unique solutions to the inverse model even with a larger number of twitch parameters. For these reasons, we used a previously proposed twitch model that included electromechanical delay, peak amplitude, time to peak, and half relaxation time (Raikova et al. 2007).

## Abbreviations

DR: Discharge rate
HDsEMG: High-density surface electromyography
MU: Motor unit
MUAP: Motor unit action potential
MVC: Maximal voluntary contraction
TA: Tibialis anterior
